# Broadband nanophotonic wireless links and networks using on-chip integrated plasmonic antennas

**DOI:** 10.1038/srep19490

**Published:** 2016-01-19

**Authors:** Yuanqing Yang, Qiang Li, Min Qiu

**Affiliations:** 1 State Key Laboratory of Modern Optical Instrumentation, College of Optical Science and Engineering, Zhejiang University, Hangzhou 310027, China

## Abstract

Owing to their high capacity and flexibility, broadband wireless communications have been widely employed in radio and microwave regimes, playing indispensable roles in our daily life. Their optical analogs, however, have not been demonstrated at the nanoscale. In this paper, by exploiting plasmonic nanoantennas, we demonstrate the complete design of broadband wireless links and networks in the realm of nanophotonics. With a 100-fold enhancement in power transfer superior to previous designs as well as an ultrawide bandwidth that covers the entire telecommunication wavelength range, such broadband nanolinks and networks are expected to pave the way for future optical integrated nanocircuits.

Nanoantennas, optical devices that can mediate between localized energy and free-space radiation, have become a subject of great interest in recent years^1^. Their intriguing properties, which allow us to manipulate light at the nanoscale while controlling the far-field emission, have a dramatic impact on a variety of fields, including spectroscopy^2^,^3^, photoemission and photodetection^4^,^5^,^6^,^7^,^8^,^9^, sensing^10^,^11^, metasurfaces^12^,^13^, nonlinear optics^14^,^15^ and nanoscale optical circuitry^16^,^17^,^18^,^19^,^20^. In this regard, Alù and Engheta first proposed an optical wireless nanolink using matched dipole nanoantennas^16^. As an alternative to the conventional plasmonic waveguide interconnects, the optical wireless nanolink has been demonstrated to exhibit lower loss and better performance than its “wired” counterpart. Moreover, the optical wireless link can provide simpler network architectures and more on-chip space, opening new venues for upcoming nanoscale optical chips and their further miniaturization.

For general wireless communications, bandwidth is a key parameter and a determinant for the capacity of the links and the networks. Broadband wireless communications are capable of transporting signals through multiple channels within different frequency bands, thereby largely enhancing the information-carrying capacity of communication systems. However, this issue has not been investigated in the nanophotonics domain. In previous research on the optical wireless nanolinks, nanoantennas such as dipole, Yagi-Uda and phased-array configurations were applied^16^,^17^,^18^. These nanoantennas can only work at resonant wavelengths with very narrow bandwidths. Moreover, when coupled to waveguides, there exists large impedance mismatches between the resonant nanoantennas and the waveguides. Impedance matching elements such as optical nanodisks of certain permittivities are needed to be carefully designed and positioned within the connecting gap between the nanoantenna and the waveguide^16^,^17^, which is neither flexible nor easy to control with current nanofabrication techniques.

In this paper, we transplant the concept of broadband wireless communications into the nanophotonics community. More specifically, we show the complete design of broadband optical wireless nanolinks and functional networks. To this end, plasmonic horn nanoantennas which have been proposed in our previous study^7^ are adapted and utilized as basic building blocks, since they can be readily fabricated and naturally impedance-matched to the feeding waveguides. We demonstrate that, the non-resonant behavior, arising from the absence of the resonant elements, allows the horn nanoantennas to operate over a wide range of frequencies with high directivities and high gains. A point-to-point optical wireless nanolink using the horn nanoantennas thus shows a significantly superior performance (100-fold enhancement in power transfer at telecommunication wavelengths and a much wider bandwidth) to that using dipole nanoantennas. Based on the broadband nanolink, we further demonstrate a broadcast optical wireless network, including a transmitter, a wavelength-division multiplexing (WDM) wireless router and receivers. The concept and complete design of such broadband optical wireless nanolinks and networks are expected to offer new possibilities for future chip-scale photonic nanocircuits.

## Results and Discussion

### Plasmonic horn nanoantenna

**Figure 1:**
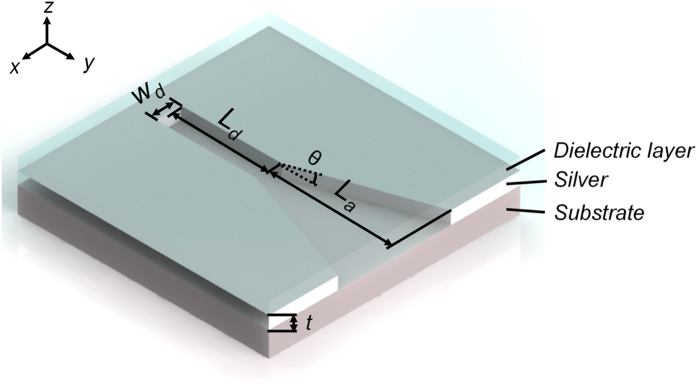
The schematic diagram of a plasmonic horn nanoantenna. (A) dielectric cladding layer are used to realize a homogeneous environment to provide a homogeneous environment.

To implement optical wireless nanolinks and networks, first we need to choose suitable nanoantennas functioning as basic building blocks: the transmitter and receiver. Here we employ plasmonic horn nanoantennas, as sketched in Fig. 1. Plasmonic channel waveguide, a rectangular slot of width *W*_*d*_ = 30 nm carved in a silver film of thickness *t* = 50 nm, is used as an optical transmission line. The length of the waveguide is fixed as *L*_*d*_ = 500 nm. The horn nanoantenna with length *L*_*a*_ and flare angle 2θ appears like a flared-out waveguide. Substrate and dielectric cladding layer covering the whole structure are assumed to possess a refractive index of *n* = 1.5.

The horn nanoantennas are investigated by performing numerical finite-difference-time-domain (FDTD) simulations (FDTD Solutions, Lumerical). To give a clear illustration of how the plasmonic horn nanoantennas work and reveal the underlying physics, we start our investigation at a given wavelength λ_0_ = 1550 nm. The optical constants of silver used in the calculation are obtained by fitting the values found in Ref. 21 to a mutilcoefficient model. The quasi-TEM mode^22^ supported by the plasmonic slot waveguide is calculated and then employed as an integrated source to excite the whole system. It is worth noting that the structure shown here is different from what we have proposed in Ref. 7. In the previous design, the plasmonic waveguide consists of two parallel wires with rectangular cross sections. The edge of the waveguide has a finite width of 50 nm, in which case two or more propagating modes would exist simultaneously^7^,^23^. However, in our current design, the edge of the waveguide is assumed to be long enough and infinite at *x* direction, ensuring only asymmetric mode^23^ is supported inside the gap and providing a further convenience for on-chip integration. Also, the geometric difference has an impact on the far-field radiation characteristics, which we will discuss later.

**Figure 2:**
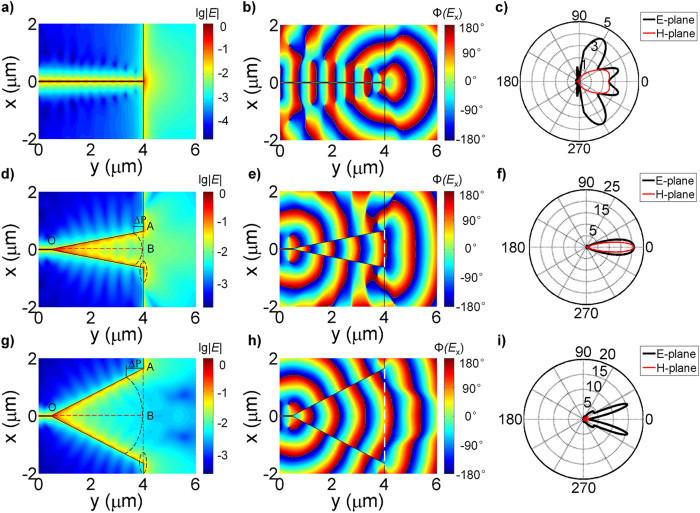
Near-field profiles and far-field radiation patterns of a bare waveguide and horn nanoantennas with different flare angles. (**a**–**c**) Bare waveguide case. The amplitude (**a**), phase (**b**) of electric field distribution and radiation pattern (**c**) of a bare waveguide. (**d**–**f**) A horn nanoantenna with *L*_*a*_ = 3.5 μm and θ = 10°. The amplitude (**d**), phase (**e**) of electric field distribution and radiation pattern (**f**). (**g**–**i**) A horn nanoantenna with *L*_*a*_ = 3.5 μm and θ = 25°. The amplitude (**g**), phase (**h**) of electric field distribution and radiation pattern (**i**).

For a bare waveguide, most of energy is reflected at the open end and cannot radiate efficiently, as reported previously^17^,^20^ and shown in Fig. 2a. The phase distribution in Fig. 2b implies the radiation from the open-ended waveguide mimics a unidirectional point source with a spherical phase front, accompanied by phase discontinuities on two sides. The far-field radiation pattern displayed in Fig. 2c further verifies this point. A low directivity of around 2 is produced by the unidirectional spherical waves and the large side lobes are due to the phase discontinuities and residual radiation from the two sides. Here the directivity is defined as *D*(φ,ϕ) = 4π*p*(φ,ϕ)/*P*_rad_ in a linear scale, where φ and ϕ represent the direction of the observation, *p*(φ,ϕ) is the angular power density and *P*_rad_ is the radiated power. In comparison with the bare waveguide, a horn nanoantenna with a tapered-out aperture provides a gradual and smooth transition from subwavelength-localized energy to the free-space waves, as shown in Fig. 2d. With the increase in the slot width, the confined plasmon mode gradually becomes decoupled plasmon modes propagating along the surfaces of the silver plates and finally radiates into the free space through the horn aperture. A large aperture with a relatively uniform phase front resembles a plane-wave source and thus can produce a highly directional radiation, as suggested in Fig. 2e,f. However, if the horn aperture continues increasing, the field and phase distributions across the horn aperture become far away from being constant, resulting in the split of the radiation pattern and remarkable reduction in the directivity along the horn axis.

The phase variation of the field across the aperture can be illustrated by applying a simple geometrical model. Referring to Fig. 2d,g, we assume an imaginary line source exists at the apex of the horn nanoantenna (point O). The constant phase fronts of the radiating waves are cylindrical. Since there is a path difference ΔΡ between the waves travelling along the side and the axis of the horn, the phases at point A and point B at the aperture of the horn are different. Additionally, distinct from radiofrequency horn antennas where metals are treated as perfect electric conductors, here an abrupt phase change Δφ occurs outside the aperture of the plasmonic horn, due to the reflection and scattering of the propagating plasmons. This phase change, shown by the dashed circle in Fig. 2d,g, also contributes to the far-field radiation. Hence, the phase variation δ_0_ for a plasmonic horn nanoantenna can be written as:


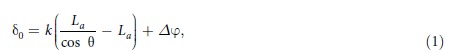


where *k* is the wave number of the propagating waves inside the horn nanoantennas. To make the phase variation as small as possible, a short length and a small flare angle are preferred. However, as discussed above, a large aperture (i.e., the length and the angle could not be too small at the same time) is needed to provide the gradual impedance matching and thus ensures the efficient radiation. Therefore, a trade-off between these extremes should be taken into account in practical applications. A systematic study on the performance of the nanoantennas with respect to the geometric parameters is necessary.

### Radiation characteristics

**Figure 3:**
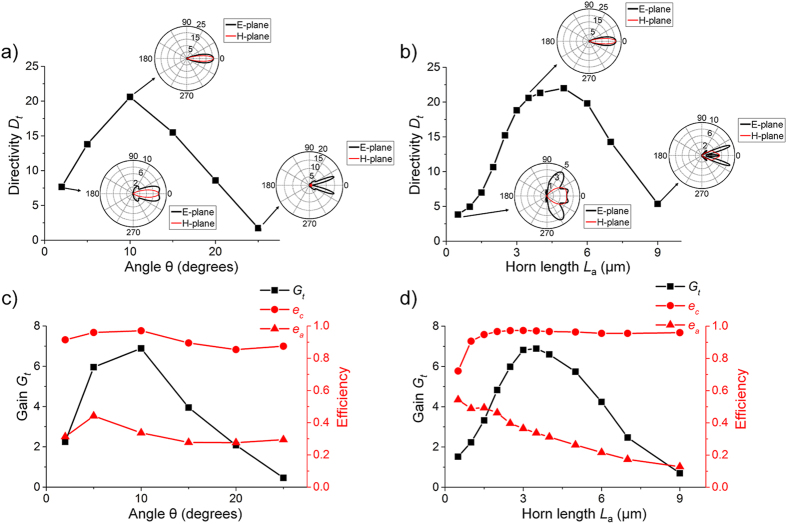
Radiation characteristics as functions of geometric parameters. Directivity *D*_*t*_ and gain *G*_*t*_ as well as coupling and radiation efficiency for plasmonic horn nanoantennas with a fixed length *L*_*a*_ = 3.5 μm and varying angles (**a,c**) or with a fixed angle θ = 10° and varying length *L*_*a*_ (**b,d**). The insets in (**a,b**) show the corresponding radiation patterns of nanoantennas with different geometric parameters.

The directivity along the axis of the horn, denoted as *D*_*t*_, is plotted in Fig. 3a,b as a function of flare angle and antenna length. For a given length *L*_*a*_ and increasing small angles θ, the E-plane pattern becomes narrower and *D*_*t*_ becomes larger. However, beyond a certain angle, *D*_*t*_ begins to decrease as the flare angle continues increasing, indicating the shrink of the main lobe. Eventually, the pattern splits and the main maximum does not even appear on the axis. This variation is in accordance with the discussion in Fig. 2 and can also be observed for a constant angle and varying lengths, as displayed in Fig. 3b. We note that the relation between the directivity and the geometric parameters is also consistent with our previous study^7^, further proving the validity of above discussion on the phase variation. Nevertheless, current structure possesses a higher directivity ( > 20) than the previous one (~13) due to the geometric differences.

Besides directivity, other parameters such as coupling efficiency *e*_*c*_, radiation efficiency *e*_*a*_ and gain *G*_*t*_ are also important measures for antennas used in wireless communications^1^,^24^. The coupling efficiency *e*_*c*_ describes the mismatch in feeding a nanoantenna and is defined as *e*_*c*_ = 1-|Γ_t_|^2^, where Γ_t_ is the voltage reflection coefficient at the connection point between the waveguide and nanoantenna. The radiation efficiency *e*_*a*_ illustrates the capability of a nanoantenna as a transducer between the guided energy and free-space radiation. It can be expressed as *e*_*a*_ = *P*_rad_/*P*_*t*_, where *P*_*t*_ is the power delivered to the nanoantenna from the waveguide. The gain *G*_*t*_ = *e*_*c*_*e*_*a*_*D*_*t*_ is an overall figure of merit that takes into account both the antenna efficiency and its directional properties.

For the coupling efficiency *e*_*c*_, since the horn nanoantenna is inherently connected to the waveguide, it generally stays high with varying geometric parameters, as suggested in Fig. 3c,d. In most cases, the coupling efficiency *e*_*c*_ is higher than 90%. Only for horns with short lengths (*L*_*a*_ < 1 μm), the coupling efficiency *e*_*c*_ becomes notably lower. This is because the small aperture of a short horn, which approximates an open-ended waveguide, is unable to afford a gradual transition for impedance matching, thereby decreasing the coupling efficiency. While the coupling efficiency *e*_*c*_ is relatively stable, the radiation efficiency *e*_*a*_ shows a strong dependence on the horn length *L*_*a*_. Longer horns bring in higher ohmic loss and thus lead to lower radiation efficiency. Different from the efficiencies, the gain *G*_*t*_, however, exhibits optimal values as functions of horn length and angle, indicating that an optimum horn nanoantenna can be realized within a given range of dimensions. In fact, from a practical point of view, the dimensions of the horn always need to meet some particular criteria such as a high integration density. The results are consequently expected to guide the design and facilitate the application of the horn nanoantennas.

### Broadband features

**Figure 4:**
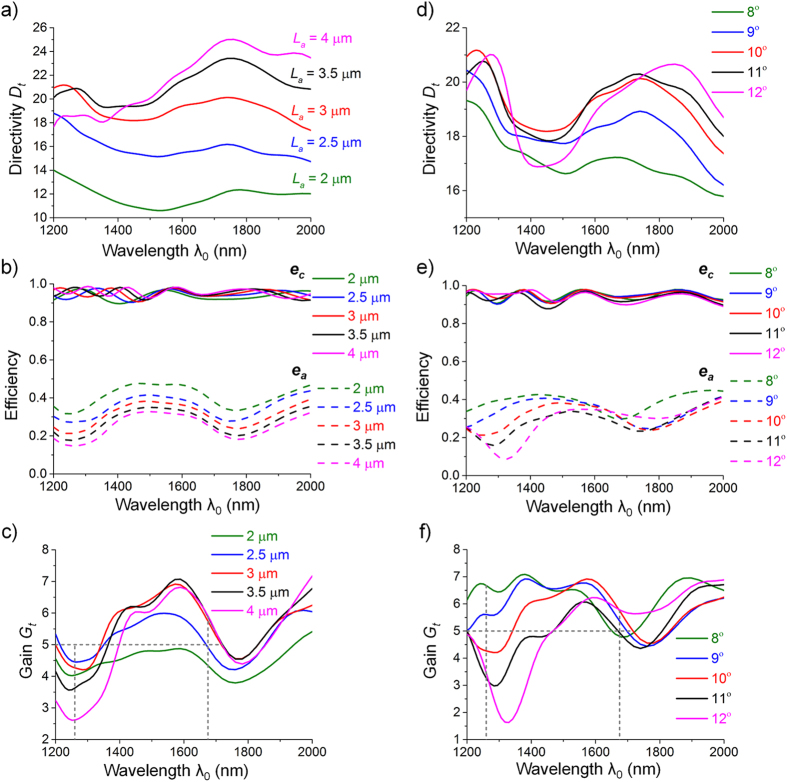
Broadband properties of the horn nanoantennas. The broadband response with variations in horn length (**a–c**) and angle (**d–f**) are plotted. The gray dashed lines in Fig. (**c**) and (**f**) indicate the wavelength range from O band to U band (1260 – 1675 nm) as well as the specific standard, *G*_*t*_ = 5, for determining the bandwidth.

Noticing that the above discussion is based on a single-frequency situation, we further study the broadband characteristics of the horn nanoantennas, as shown in Fig. 4. The variation range of the geometric parameters is given around the optimal values in the single-frequency case (i.e., *L*_*a*_ = 3.5 μm and θ = 10°). The directivity *D*_*t*_ generally increases with the dimensions and keeps larger than 10 over a broad wavelength range from 1200 nm to 2000 nm. The coupling efficiency *e*_*c*_ stays high and the radiation efficiency *e*_*a*_ decreases as the length and angle grow, in a similar manner to the aforementioned single-frequency case. Here we concentrate on the telecommunication wavelengths ranging from O band to U band (1260 – 1675 nm) and set a specific standard (*G*_*t*_ > 5) to define the useful bandwidth, as indicated in Fig. 4c,f. As the horn length *L*_*a*_ increases, the bandwidth first increases and then declines with a maintained peak frequency. At the same time, the increase in the horn angle θ leads to redshifts of the spectral response. A horn nanoantenna with length *L*_*a*_ = 3 μm and θ = 9° exhibits the widest bandwidth that covers the whole wavelength range of interest with an average gain *G*_*t*_ of more than 6. This broadband response is attributed to the absence of resonant elements in the horn, making the nanoantenna a non-resonant low-*Q* radiator with impedance that remains nearly constant over a wide frequency band. By contrast, resonant nanoantennas such as dipole nanoantennas resemble relatively high-*Q* resonators with impedances changing rapidly with frequency, resulting in narrow bandwidths^16^,^20^. The superior broadband feature as well as the high gain enables the horn nanoantennas to function as pervasive building blocks for future photonic nanocircuits.

### Point-to-point broadband optical wireless nanolink

**Figure 5:**
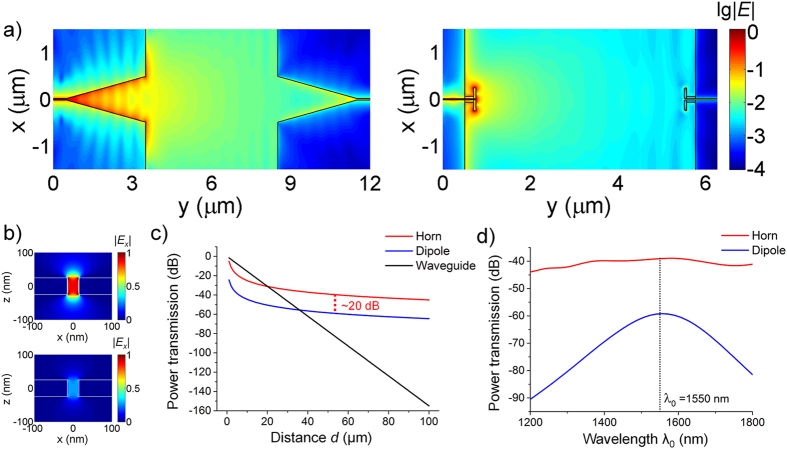
Comparison between the optical wireless nanolinks using horn and dipole nanoantennas. (**a**) The electric-field distributions of the nanolinks at λ_0_ = 1550 nm based on FDTD simulation (**b**) Corresponding E-field profiles inside the receiving waveguides of the nanolinks using horn (up) and dipole (down) nanoantennas. Two figures share the same color bar. (**c**) Power transfer of the nanolinks using horn (red) and dipole (blue) nanoantennas as well as the direct waveguide interconnect (black) at the wavelength λ_0_ = 1550 nm. The results are obtained by the Friis Equation. (**d**) Spectral power transfer of the horn- (red) and dipole-based (blue) nanolinks at *d* = 50 μm.

Next, we build a complete point-to-point optical wireless nanolink using the proposed horn nanoantennas. For simplicity, identical horns (*L*_*a*_ = 3 μm and θ = 9°) are utilized in both transmitting and receiving ends, as displayed in Fig. 5a. Another wireless nanolink based on the dipole nanoantennas is also presented as a comparison. The dipole nanoantennas are designed to be perfectly matched to the feeding waveguide and resonant at λ_0_ = 1550 nm^16^. Compared with the dipole-based nanolink, the horn-based link exhibits a pronounced enhancement in the *E*-field amplitude. Figure 5b depicts the field profile inside the waveguides at the receiving ends, confirming the plasmon modes supported by the waveguides are successfully excited. Additionally, it also demonstrates the advantage of the horn-based nanolink in wireless power transfer. To explicitly illustrate the issue of power transmission, we adopt the well-known Friis Transmission Equation^16^,^24^:


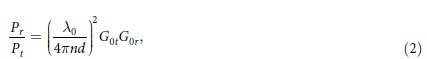


where *P*_*t*_ and *P*_*r*_ are the input and received power at transmitting and receiving terminals, *d* is the distance between the terminals, *G*_0*t*_ and *G*_0*r*_ are the gains of the transmitting and receiving antennas, respectively. Based on the Friis Equation, we compare the power transmission in three scenarios: the horn-based nanolink, the dipole-based nanolink and the direct waveguide interconnect, as shown in Fig. 5c. At wavelength λ_0_ = 1550 nm, the horn nanoantenna possesses a high gain *G*_*t*_ of 6.8 with a directivity *D*_*t*_ close to 20 while the gain of the dipole nanoantenna only reaches 0.66 with a low directivity around 1.5^16^,^17^. A 100-fold enhancement in the wireless power transmission is thus achieved. Meanwhile, at large distances of several tens of microns, both the horn- and dipole-based wireless nanolinks show significantly lower loss than the direct waveguide link, as reported in previous studies^16^,^17^. The spectral power transmission can also be calculated by using equation (2), as shown in Fig. 5(d). The wireless power transmission via the horn-based nanolink is relatively stable with only small variations over the entire wavelength range of interest, thus showing an evident broadband features. Conversely, the wireless power transmission via the dipole-based nanolink exhibits a large fluctuation of more than 30 dB in the telecommunication wavelength range, verifying the narrow bandwidth of such nanolink.

### Functional optical wireless network

**Figure 6:**
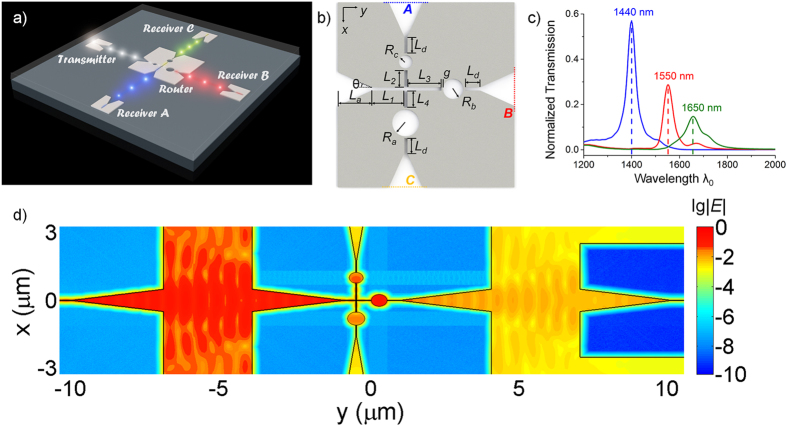
Broadband optical wireless network. (**a**) The schematic diagram. White color represents the broadband signal while other colors indicate different channels. The whole network is embedded in a homogenous environment. (**b**) The sketch of the WDM wireless router. A, B, C are three different output ports. (**c**) Normalized power transmissions at different output ports of the router. (**d**) E-field distribution of the wireless network at λ_0_ = 1550 nm.

The wide bandwidth of the horn-based point-to-point links lays the foundation for broadband optical wireless communications at the nanoscale. Here, by integrating with other plasmonic nanodevices, we introduce the concept and design of broadband optical wireless networks. As a simple example, a broadcast network with star topology is presented, as shown in Fig. 6a. The network contains a transmitter, a wireless router and three receivers. A broadband signal sent by the transmitter is first delivered to the wireless router and then separated into three different channels with different wavelengths. After that, the router directs the divided signals in different directions and guides them to different receivers through the horn nanoantennas. Thus, the receivers can obtain the signals within specific wavelength bands on demand. In such a broadcast network, the wireless router is the most important element. To achieving the WDM and split functions, we exploit the plasmonic splitters^22^ and nano-disk cavities^25^ as well as the horn nanoantennas in the design of the router, as depicted in Fig. 6b. For optimizations of the wireless router and network, we employ 2D FDTD simulation due to the computer memory and calculation time constraints. The thickness of the metal is assumed to be infinite. Geometric parameters are chosen as follows: *L*_*a*_ = 3 μm, θ = 9°, *L*_1_ = *L*_3_ = *L*_4_ = *L*_*d*_ = 0.5 μm, *L*_2_ = 0.75 μm, *R*_*a*_ = 0.29 μm, *R*_*b*_ = 0.265 μm, *R*_*c*_ = 0.235 μm, *g* = 0.01 μm. The output power at different ports of the router is displayed in Fig. 6c, indicating a power ratio of 4:2:1 for the three channels. We note that, by adjusting the geometric parameters, the power ratio can be tuned to satisfy other specific requirements^26^. The near-field distributions of the network is also shown in Fig. 6d, indicating the signal in the channel of 1550 nm is successfully sent through the right path and finally arrives at the matching receiver. Last but not least, although the presented WDM wireless network has a simple topology and functionality, it provides a new paradigm for broadband optical wireless communication at the nanoscale. Many other types of nanoantennas or nanodevices such as isolators, switches can also be added to functionalize such broadband wireless network.

## Conclusion

Broadband wireless communications are of central importance at radio and microwave frequencies. In this paper, we realized the powerful concept in the nanophotonics regime. Specifically, we demonstrated the complete design of efficient broadband optical wireless nanolinks and networks. As their fundamental building blocks, on-chip integrated plasmonic horn nanoantennas are studied thoroughly. With a 100-fold enhancement in the wireless power transfer and an ultrawide bandwidth that covers the whole telecommunication wavelength range, the broadband nanolinks and networks largely outperform the previous designs as well as the direct waveguide interconnects, opening the prospect of high-capacity optical wireless communications at the nanoscale.

## Methods

**Figure 7:**
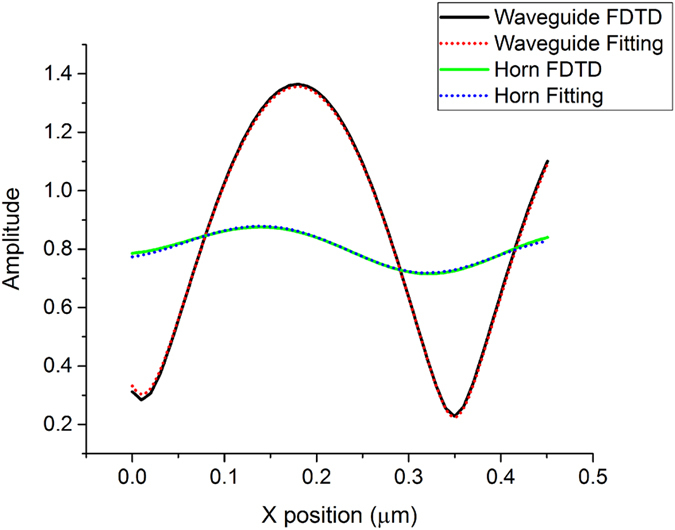
The near-field intensity profiles and nonlinear-fitting curves for a bare waveguide and a horn nanoantenna with *L*_*a*_ = 3.5 μm and θ = 10°.

FDTD simulations are performed using a commercial software FDTD Solutions v8.11.318, Lumerical. For all calculations, perfectly matched layers (PMLs) are applied to enclose the simulation area. Finest mesh grid size of 2 nm are used in the simulations. In both 3D and 2D simulations, we calculate the quasi-TEM mode directly by using the optical mode solver integrated in the FDTD Solutions and apply the mode as an integrated source to excite the nanoantennas and systems. At λ_0_ = 1.55 μm, it has a propagation constant γ_0_ = 0.1784 + 9.1613 *j* μm^-1^, corresponding to a propagation loss of 1.54 dB/μm. The pure input power *P*_*t*_ thus can be simply obtained as 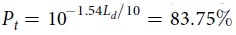 normalized to the initial power of the mode source. To derive the coupling efficiency *e*_*c*_ and the reflection coefficient Γ_t_, we record the near-field intensity inside the gap and apply a nonlinear fitting procedure^17^,^20^. The field amplitude inside the gap at position *x* could be written as 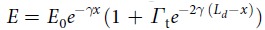, where *E*_0_ is the maximum field amplitude, γ is the propagation constant. Figure 7 depicts the near-field intensity profiles and their fitting curves for a bare waveguide and a horn nanoantenna with *L*_*a*_ = 3.5 μm and θ = 10°. A strong standing wave pattern could be clearly observed for the bare waveguide and the reflection coefficient could be determined as Γ_t_ = 0.7523*e*^−0.3505*j*^, meaning that 56.6% of the power transmitted to the waveguide is reflected. While for the horn nanoantenna, Γ_t_ = 0.092*e*^0.2364*j*^, indicating more than 99% of the input power could be transferred to the horn nanoantenna. In addition, the propagation constant could also be derived via the nonlinear fitting of the curves as γ = 0.1722 + 9.266 *j* μm^−1^, agreeing very well with the value γ_0_ that is obtained from the mode solver directly. The radiated power *P*_rad_ is attained by integrating the outward Poynting vector over a closed box that encloses the horn nanoantenna but excludes a small rectangular area where the waveguide enters the box. The directivity and radiation patterns are obtained by performing a standard near-to-far-field projection of the time-averaged fields recorded in the closed box. For far-field projections, we calculate the far-field profile on a spherical surface which is 1 meter far away from the simulation region. The resolution of the far-field radiation patterns is 1°.

## Additional Information

**How to cite this article**: Yang, Y. *et al*. Broadband nanophotonic wireless links and networks using on-chip integrated plasmonic antennas. *Sci. Rep.* **6**, 19490; doi: 10.1038/srep19490 (2016).
